# Sleep Disturbances and Depression Levels among General Indonesian Population: A National Survey

**DOI:** 10.2174/0117450179326359240903045716

**Published:** 2024-09-11

**Authors:** Sofa D. Alfian, Jihan N. Thurfah, Meliana Griselda, Irma M. Puspitasari

**Affiliations:** 1 Department of Pharmacology and Clinical Pharmacy, Faculty of Pharmacy, Universitas Padjadjaran, Jatinangor, Indonesia; 2 Center of Excellence for Pharmaceutical Care Innovation, Universitas Padjadjaran, Jatinangor, Indonesia; 3 Center for Health Technology Assessment, Universitas Padjadjaran, Jatinangor, Indonesia; 4 Pharmacist Professional Program, Faculty of Pharmacy, Universitas Padjadjaran, Jatinangor, Indonesia

**Keywords:** Sleep disturbance, Depression, Depression levels, Center for epidemiologic studies-depression revised (CESD-R-10) scale, T-score, The indonesian family life survey (IFLS)

## Abstract

**Background:**

The correlation between sleep disturbance and depression is widely recognized in developed countries but relevant evidence is lacking in developing countries.

**Objective:**

This study aims to assess the correlation between sleep disturbance and depression levels among the general Indonesian population.

**Methods:**

This national cross-sectional survey was conducted using the Indonesian Family Life Survey. Sleep disturbance was assessed based on the questions related to sleeping experience. Depression levels were assessed with a modified Center for Epidemiologic Studies-Depression scale. Sociodemographic factors as confounders were obtained from the self-reported data. Logistic regression was performed after adjusting for confounders.

**Results:**

A total of 22,024 respondents were included. Respondents with severe, moderate, and mild sleep disturbance were associated with depression compared to those with none or slight sleep disturbance.

**Conclusions:**

Respondents with sleep disturbance had a higher possibility of experiencing depression. Screening of sleep quality among the general population is important to reduce the risk of depression.

## 1 INTRODUCTION

Sleep is a routine human activity crucial for the restoration of physical and mental conditions [1]. Considering its significance, sleep is among the three fundamental pillars of health after a healthy diet and regular exercise [2]. Good quality of sleep is essential for mental health, positive cognitive functioning and metabolic, cardiovascular, and cerebrovascular health [3]. This requires sufficient duration, regularity, appropriate timings, and absence of sleep-related disorders [4]. Sleep disorders are disturbances in the quality, amount, or sleep timing [5] caused by psychological distress [6], negative psychological reactions, trauma [7] environmental factors including noise, lights, and unusual odors, as well as medical interventions [8]. A previous report stated that 16.6% of 43,935 individuals across Ghana, Tanzania, South Africa, India, Bangladesh, Vietnam, Indonesia, and Kenya had severe or extreme nocturnal sleep problems [9]. Additionally, in Indonesia, sleeping disturbances have been reported to affect 28 million individuals, or approximately 10% of the total population [10]. Another study conducted among 10,132 individuals across the USA, Western Europe, and Japan showed sleep disturbance prevalence of 56%, 31%, and 23% [11]. In the United States, 14.5% of adults were recorded to experience trouble falling asleep [12], while approximately 48% of the adult population had at least two sleep-related issues in Australia [13]. In South Africa, the total prevalence of insomnia symptoms among 15,133 individuals was 7.1% [14]. The existence of disturbances in sleep can increase the risk of cardiovascular or infectious diseases, cancer [15], as well as mental health issues, such as depression, bipolar, and anxiety disorders [16]. The relationship between sleep disturbance and depression remains reciprocal, as severe sleep disturbance often presents with depressive symptoms [17]. Meanwhile, it has been established that depressed individuals are more prone to insomnia symptoms [18]. An estimated 3.8% or approximately 280 million of the worldwide population experience depression [19]. In Indonesia, a prevalence of 6.1% was reported in 2018, predominantly consisting of individuals aged >75 years [20]. Depression can increase multimorbidity risk [21] and suicidal ideation [22]. According to the World Health Organization [23], the frequency of suicide is >700,000 annually, ranking fourth as the leading cause of death in the world among individuals in the 15-29 years of age group [19]. In Indonesia, the age-standardized suicide rate per 100,000 individuals of all ages was reported as 2.6 by WHO in 2021 [23]. Moreover, the suicide rate in 2022 at 826 cases was higher than the 613 and 670 cases reported in 2021 and 2020 [24]. The underreporting rate of suicidal cases was 303%, which was significantly higher compared to the global average [25], indicating a possible absence of more reported cases. Therefore, the high rate of depression cannot be underestimated, posing a potentially life-threatening risk to affected individuals. The increasing rates of depression among the elderly in Indonesia (16.3% of 4,236 in 2014 to 11.2% of 28,570 in 2018) [20, 26] and young adults aged 18-24 years old (27.8% of 1960 in 2014 to 5.6% of 37,243 in 2018) [20, 27]. Furthermore, a study revealed that 5.1% of 26,936 individuals aged 15-17 years old exhibited depression symptoms in 2018 [20, 28]. At the same time, Indonesian populations reported a high prevalence of sleep disturbance, with 33.3% of 31,432 individuals aged 15 years and above experiencing sub-threshold insomnia and 11.0% exhibiting clinical insomnia symptoms [29]. The correlation between sleep disturbance and depression is widely acknowledged in developed countries [30], but there is limited information in developing countries, including Indonesia. Previous studies were conducted only on a small scale of respondents [9], focusing on older age group [31], or using outdated sources of data [31, 32]. A similar investigation was conducted using the IFLS-5 as a national data source, but without comprehensively assessing the total quality of sleep disturbance [33]. Therefore, this study aimed to assess the correlation between sleep disturbance and depression levels among the general population in Indonesia.

## 2 MATERIALS AND METHODS

The present investigation was carried out in compliance with the cross-sectional study principles outlined in the Strengthening the Reporting of Observational Studies in Epidemiology (STROBE) [34].

### 2.1 Design of Study and Source of Data

The fifth wave of the national longitudinal data from the Indonesian Family Life Survey (IFLS-5) was used for this observational cross-sectional investigation. The data were gathered between 2014 and 2015, and were made publicly available in 2016 (https://www.rand.org/well-being/social-and-behavioral-policy/data/FLS/IFLS/access.html). The IFLS is a nationwide longitudinal study that represents around 83% of Indonesians through the use of a multistage stratified sample design, including over 50,000 individuals from 13 out of 33 provinces [35]. Implemented by the RAND Corporation in 1993, the IFLS-5 is a continuous health and socioeconomic survey that is performed at individual, family, and community levels. The IFLS sampling approach used random selection within strata based on provinces and urban/rural areas. Given the size and topography of the nation, provinces were chosen to maximize population representation, reflect the diversity of Indonesian culture and socio-economic status, and assure cost-effectiveness. As a result, 13 of the 27 provinces that were in existence at the time were covered. In order to enable comparisons between urban and rural areas as well as between Javanese and non-Javanese, the IFLS randomly chose 321 enumeration areas (EAs) within each of the 13 provinces, exceeding the sample size EAs in less populous provinces and EAs in metropolitan areas. There were thirty homes chosen from the rural EA and twenty from every urban EA [35]. In addition, the information gathered comprised assessments of biomarkers, self-reported medical history, symptoms, and aches, as well as sociodemographic, economic, and medical status variables.

Before the full-scale survey was conducted, the first evaluation of the IFLS questionnaire was carried out to ensure reliability and validity [35].

### 2.2 Study Population

In this study, respondents aged at least 18 years during data collection and those with complete information were included. The respondents whose data were not available about their sleeping experience and depression levels were excluded.

### 2.3 Sleep Disturbance

Sleep disturbance was assessed based on responses to a 10-question sleeping experience questionnaire (Table **S1**). The questionnaire was validated and adopted from the Patients-Reported Outcomes Measurement Information System (PROMIS) [36]. The PROMIS questionnaire passed through an initial translation into Indonesian (forward translation). Subsequently, two translators performed a distinct reverse translation into English [37]. Each question was related to the respondents’ sleeping experience in the past 7 days and graded according to a five-point scale, as follows: 1, 2, 3, 4, and 5 for never, rarely, sometimes, often, and always, respectively (score range:10–50) [36]. The severity of sleep disturbance was greater as indicated by higher scores [36]. The score obtained from the questionnaire was defined as a raw score, which was converted to a T-score using the following formula:


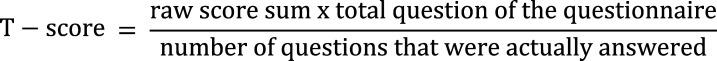


T-score was grouped to categorize the level of sleep disturbance as follows: <55 = none to slight sleep disturbance, 55.0–59.9 = mild sleep disturbance, 60.0–69.9 = moderate sleep disturbance, ≥70 = severe sleep disturbance [38].

### 2.4 Sociodemographic Characteristics

The sociodemographic factors, as potential confounders, were evaluated using information from a self-reported survey. These factors consisted of age groups (18–29, 30–49, and >49 years), gender (male/female), body mass index (lack of nutrients, underweight, normal, overweight, and obese), marital status (unmarried, married, legally divorced, and widow(er)), working status in the past 12 months (employed/unemployed), level of education (elementary, middle, high school, and university), and location of residence (rural/urban). The variables were selected to estimate the direct and total effects of the exposure to the outcomes [39]. Based on previous studies showing significant effects of some socio-demographic factors on sleep quality [40-42], these socio-demographic variables were included as confounding variables.

### 2.5 Depression

The symptoms of depression were assessed using 10 items from the Center for Epidemiologic Studies Depression scale revised (CESD-R-10), reflecting the experiences of respondents in the last week (Table **S2**). Respondents rated their feelings from rarely or never (≤1 day), to some days (between 1–2 days), occasionally (3–4 days), and most often (5–7 days), equivalent to scores 1, 2, 3, and 4. A total score of ≥10 suggested that depressed symptoms were present [43]. First, a forward translation into Indonesian was used for the CESD-R-10 questionnaire and subsequently translated again into English by two different individuals (reverse translation) [37]. Generally, the CESD-R-10 scale is commonly used to measure depression symptoms due to its validated reliability in identifying individuals, who are at a potentially high risk of developing depression [43].

### 2.6 Statistical Analysis

Descriptive analyses were used to provide an overview of the respondents' characteristics. The bivariate association between the degree of sleep disturbance and depression was assessed using a Chi-square test. In the bivariate analysis, the potential factors were shown to be linked with the outcome at a significance level of p < 0.25, and were then assessed using an initial multivariate model. Complete case analyses were conducted considering that a small amount of data was missing. Subsequently, the correlation between sleep disturbance and depression levels was ascertained using multivariate binary logistic regression, after adjusting for potential confounders. The adjusted association was obtained along with an odds ratio (OR) at a confidence interval of 95%. All of the factors that were comprised in the final model had p-values fixed at 0.05. To evaluate the multivariate model, the Omnibus and Nagelkerke R-Square tests were employed. All statistical analyses were carried out using the Statistical Package for the Social Sciences (SPSS) version 26.0.

## 3 RESULTS

### 3.1 Characteristics of Respondents

Among the 50,000 respondents interviewed in the survey, 27,976 (55.95%) who did not fulfill the question- naire related to sleeping experience and depression symptoms assessment were excluded. This resulted in a total of 22,024 respondents being incorporated into this research. Most of the individuals who participated were of the age 30-49 years old (44.5%), female (53.5%), urban dwellers (59.3%), completed their primary education (31.7%), had been employed in the previous 12 months (91.1%), presently married (72.9%), and with a normal BMI (56.3%), as shown in Table **1**. A total of 7 and 1,116 data were found missing on marital status and educational level but remained in the study by employing the list wise elimination technique [44]. Furthermore, the majority of respondents who were not experiencing sleep disturbances and depression had proportions of 57.1% and 99.1%, respectively.

### 3.2 Correlation between Sleep Disturbance and Depression Levels

The multivariate analysis comprised the bivariate analysis results with p<0.25 to subsequently determine the factors playing a role in the incidence of depression (Table **S3**). In the multivariate analysis presented in Table **2**, respondents with severe (OR = 28.38; confidence interval [CI] 95% = 17.69–45.51), moderate (OR = 10.15; CI 95% = 6.68–15.43), and mild sleep disturbance (OR = 2.97; CI 95% = 1.82–4.83) showed the potential of experiencing depression. Furthermore, the Omnibus test gave p = 0.000, indicating that the multivariable analysis model was feasible and fulfilled the requirement. The results of the Nagelkerke R-Square test showed that the factors in the model explained depression by 12.5%.

## 4 DISCUSSION

Among the 22,024 respondents, the majority reported no experience of sleep disturbance or depression. After adjusting for confounding variables, a significant association was observed between sleep disturbance and an increased risk of depression. These results were consistent with past reports showing an association between a worse quality of sleep and a higher risk for depression [45]. The significant association may be explained by altered neurological functioning [46], problems in regulating behavior [47], and deprived emotional expressiveness [48]. Furthermore, a previous study showed an association between shortened sleep duration and worsened emotional regulation among young adolescents [49], as a risk factor for major depressive disorders [50]. Longitudinal analyses showed that mal- adaptive emotional regulation played a significant role in mediating the relationship between sleep and depression levels [30]. Therefore, impaired emotional regulation showed the potential to alter the neural processes, resulting in the symptomatology of depression [51].

Impaired emotional regulation due to sleep disturbances can originate from aberrations in neuro- transmitters and brain structures in the problems of sleep–wake cycle, depression, and anxiety. Additionally, biological factors such as high inflammatory dysregulation are correlated with depression as a response to sleep disturbances [52]. A proposed mechanism for elucidating how poor sleep quality increases depression risk is an increase in the expression of inflammatory markers, such as C-reactive protein and interleukin-6 (IL-6). These markers are indicators of low-grade inflammation, a risk factor for the development of depression [53]. Elevated IL-6 levels have been observed in individuals with depressive disorders, impacting serotonin metabolism within the central nervous system [53].

An association has been established between sleep disturbances and depression levels (mild, moderate, severe), bridged by the inclusion of serotonin and melatonin hormones [54], which is known as a sleep hormone. Neurotransmission of serotonin (5-HT) in the brain regulates emotions, indicating that its deficits are associated with the pathophysiological aspects of major depressive disorder [55]. When the serotonin hormone level is low, the melatonin decreases, leading to sleep disturbances [55, 56] and causing insomnia [57].

Based on the theory above, it has been established that the lack of sleep correlates to the increasing inflammatory cytokines (IL-6 and TNF) in the body [58-63]. Meanwhile, there is a significant relationship between inflammation markers and depression [62]. Inflammation markers were higher in individuals with depression compared to those in the non-depressed group [16]. This indicates that longer episodes of insomnia affect depression and/or impair treatment response to antidepressants [57, 64]. Further- more, respondents with lower quality of sleep showed an increment of approximately 30% to 70% higher in the prevalence of mild, moderate, and severe depression compared to those with good sleep quality [65]. Sleep quality is described as the satisfaction of individuals [66] and not based on duration. Similarly, a prior study found a U-shaped relationship between depression and sleep length. The results showed that shorter and longer sleep duration had 121% and 97% increased risk of developing depression, respectively, compared to normal duration [67].

Previous studies suggested that factors such as older age, female gender, single status, lower education background, lower wealth status, lower income, or unemployment could influence the correlation between sleep disturbance and depression [33]. Reports indicate that individuals over 26 years old experience a deficiency in sleep quality [17]. Entering the productive age may result in an individual taking on more responsibilities, which can lead to sleep problems. Similarly, diminished body system functionality in the elderly may cause sleep problems [68]. Previous studies have identified women as unique factors that contribute to depression and sleep disturbance [17, 28]. This is due to their increased susceptibility to various factors such as emotional reactions, puberty, genetics, cyclic hormones, and sensitivity to cognitive factors such as thinking style and awareness of people's judgments [69, 70]. This phenomenon is particularly prevalent in Indonesia, where reports of gender inequality gaps are high. The single/ unmarried/divorced population was also frequently found to have a higher tendency to develop depression related to sleep disturbance [17, 33, 71]. Many studies have linked greater sleep quality to intimacy in human relationships, particularly when the closest person (husband or wife) provides positive support [45, 72, 73]. Studies have also linked poor sleep quality to a low wealth index, which is typically associated with lower education levels, lower employment status, or unemployment [17, 33]. Prior research revealed that Indonesians with more privileged educational backgrounds tend to be wealthier than their counterparts [74]. Higher education enables access to a better job and income, which provides a sense of security in life and has the positive impact of better sleep [70]. The urban residency also has its own potential influence to reduce sleep quality, which may arise from noise exposure and annoyance [75-77].

The cumulative results of this study contribute to the current knowledge about patient-related factors influencing the occurrence of depression. Therefore, screening the quality of sleep among the general population is essential to reduce the risk of depression. Several studies have reported effective and feasible interventions, such as behavior change methods, mind-body exercise [78], and cognitive behavioral therapy for insomnia, showing improvements in sleep quality (CBT-I) [79]. Other interventions, including sleep education [80], relaxation technique [81] aromatherapy, and massage [82], have been reported but their effectiveness remained inconsistent [78]. Among the existing non-pharmacological efforts for sleep quality improvement, CBT-I is widely recognized as the most effective [79, 83]. Cognitive behavioral therapy is conducted by assessing negative thoughts and providing talk therapy with a psychologist to help individuals manage problems by positively shifting their thoughts and behavior [84]. A study on the application of CBT in Indonesia showed significant improvements in total Insomnia Severity Index score [79], regulation of anxiety and low self-esteem [85], and depression level [86]. However, considerations are essential for continuous one-by-one therapy sessions and sufficient professional psychologists during implemen- tation. Another report also found that supplementation of polyunsaturated fatty acids–PUFA (eicosapentaenoic acid and docosahexaenoic acid combination), vitamin D, and probiotics could potentially be effective in reducing depressive symptoms [87] and improving sleep quality.

Interventions aimed at enhancing sleep quality and taking into account an individual's demographic background are crucial in preventing depression. Since the stigma of depression and accessing mental health services in Indonesia are still considered taboo, raising the self-help attitude by improving good mental health literacy among the public may be necessary to prevent depression related to deprived sleep quality [77]. Previous studies reported that facilitating access to mental health services through national health insurance in Indonesia helps reduce barriers to sleep disturbance [77, 88]. Further- more, encouraging the public to pay attention to their sleep could be achieved by increasing awareness of its importance and performing an initial screening of one’s sleep quality [78].

This is the first study to assess the correlation between sleep-related issues and depression levels in the general population of Indonesia through a population-based national survey. A significant strength of this study is the use of IFLS data, with a 6% dropout rate, accounting for over 83% of Indonesia's population. The IFLS offers a number of favorable conditions, such as representation of the Indonesian setting, large size, relative heterogeneity of samples, and cost-effectiveness compared with collecting new data. However, the limitations encountered during the analysis were mostly in relation to the methodology. As a cross-sectional design study, causal inferences were excluded in relation to the correlation between sleep disturbance and depression levels. As a complete analysis of the case, the statistical power might have been reduced. This phenomenon can cause potential bias in the estimation, resulting in overestimating or underestimating the conclusions. A wide CI value was also obtained, which implied a greater possibility of uncertainty regarding the precision of the estimated strength of association. Furthermore, there could have been a recall bias originating from the disparities in the accuracy of recalling past events as the responses were self-reported. Finally, the association of the model was low, suggesting that other unmeasured factors were contributing to the influence of depression status such as physical health-related factors [89], history of chronic disease [90], psychological factors (optimism, negative self-image, self-esteem, *etc*.) [89], physical activity [91], engaging in outside activity [92], usage amount of social media [93], and screen time [94]. Moreover, the recent impact of COVID-19 might influence the association between insomnia symptoms and anxiety and depression [95, 96], requiring consideration as a potential contributing factor.

Future studies should take the sampling proportions into account in the data analysis incorporating information about the correlation between sleep disturbance and depression in populations with variable socioeconomic and behavioral-related characteristics. More research is needed to determine depression states and patterns utilizing sophisticated analysis techniques like cluster analysis on a wider range of populations. Furthermore, it is advised that information on the association between sleep disturbance and depression symptoms be included in future research. Advanced study is also required focusing on sleep-related factors, and interventions on improving sleep quality along with the application, effectiveness, and prevention of depression.

## CONCLUSION

In conclusion, this study showed that respondents with sleep disturbances had a higher potential of experiencing depression. Therefore, screening the general population's sleep quality can reduce the prevalence or risk of depression. While considering one's demographic background, public health interventions should also focus on managing good sleep as a prevention of depression.

## Figures and Tables

**Table 1:** Respondent’s characteristics (N = 22,024).

**Characteristics**		**n**	**%**
Age (years)	18–29	7379	33.5
	30–49	9809	44.5
	> 49	4836	22.0
Gender	Female	11782	53.5
	Male	10242	46.5
Body Mass Index	Malnutrition	988	4.5
	Underweight	1811	8.2
	Normal	12392	56.3
	Overweight	2680	12.2
	Obesity	4153	18.9
Marital Status	Unmarried	4211	19.1
	Married	16060	72.9
	Divorced	601	2.7
	Divorced by death	1145	5.2
	*Missing*	7	0.0
Working Status	Unemployment	20065	91.1
	Employment	1959	8.9
Education Level	Elementary School	6989	31.7
	Junior High School	4232	19.2
	Senior High School	6730	30.6
	University	2957	13.4
	*Missing*	1116	5.1
Residence Location	Rural	8963	40.7
	Urban	13061	59.3
Sleep Disturbance	Severe	671	3.0
	Moderate	3642	16.5
	Mild	5141	23.3
	None to slight	12570	57.1
Depression	No	21828	99.1
	Yes	196	0.9

**Table 2:** Association between sleep disturbance and the prevalence of depression.

**Variable**	**Depression**		
	**cOR** **(95% CI)**	**aOR** **(95% CI)**	**p-value**
**Sleep disturbance**			
Severe	29.333 (18.316–46.977)	28.381 (17.697–45.516)	0.000*
Moderate	10.109 (6.660–15.344)	10.153 (6.680–15.432)	0.000*
Mild	2.948 (1.814–4.790)	2.974 (1.828–4.838)	0.000*
None to slight	-		

*Abbreviations: cOR = Crude Odds Ratio; aOR = Adjusted Odds Ratio that was adjusted by age, gender, marital status, educational level, and area of residency; CI = Confidence Interval. **Note: **Goodness of Fit Test (Hosmer and Lemeshow’s Test): p = 0.203; Coefficient of Determination (Nagelkerke R-Square): 0.125 (12.5%); Omnibus Test of Model Coefficients: p = 0.000.

## Data Availability

The data supporting the findings of the article is available on the RAND website at the address: http://www.rand.org/labor/FLS/IFLS.html.

## LIST OF ABBREVIATIONS

IFLS: = The Indonesian Family Life Survey
STROBE: = Strengthening the Reporting of Observational Studies in Epidemiology
OR: Odds Ratio

## STANDARDS OF REPORTING

STROBE and SAGER guidelines were followed.
