# Supplementary material for: Sleep Disturbances and Depression Levels among General Indonesian Population: A National Survey
Source: Clin Pract Epidemiol Ment Health. 2024 Sep 11;20:e17450179326359. doi: 10.2174/0117450179326359240903045716 (PMC11755380; doi:10.2174/0117450179326359240903045716)
Supplement: Supplementary file 1 — Supplementary material is available on the publisher's website along with the published article. [file CPEMH-20-E17450179326359_SD1.pdf]

Clin. Pract. Epidemiol. Ment. Health
ISSN: 1745-0179
DOI: 10.2174/0117450179326359240903045716, 2024, 20, e17450179326359
1
SUPPLEMENTARY MATERIAL
OPEN ACCESS

Sleep Disturbances and Depression Levels among General Indonesian Population: A National Survey

Sofa D. Alfian[1,2,3,*], Jihan N. Thurfah[1,4], Meliana Griselda[2] and Irma M. Puspitasari[1,2]

[1]Department of Pharmacology and Clinical Pharmacy, Faculty of Pharmacy, Universitas Padjadjaran, Jatinangor, Indonesia
[2]Center of Excellence for Pharmaceutical Care Innovation, Universitas Padjadjaran, Jatinangor, Indonesia
[3]Center for Health Technology Assessment, Universitas Padjadjaran, Jatinangor, Indonesia
[4]Pharmacist Professional Program, Faculty of Pharmacy, Universitas Padjadjaran, Jatinangor, Indonesia

© 2024 The Author(s). Published by Bentham Open.
This is an open access article distributed under the terms of the Creative Commons Attribution 4.0 International Public License (CC-BY 4.0), a copy of which is available at: https://creativecommons.org/licenses/by/4.0/legalcode. This license permits unrestricted use, distribution, and reproduction in any medium, provided the original author and source are credited.

*Address correspondence to this author at the Department of Pharmacology and Clinical Pharmacy, Faculty of Pharmacy, Universitas Padjadjaran, Jatinangor, Indonesia, Center of Excellence for Pharmaceutical Care Innovation, Universitas Padjadjaran, Jatinangor, Indonesia and Center for Health Technology Assessment, Universitas Padjadjaran, Jatinangor, Indonesia; E-mail: sofa.alfian@unpad.ac.id

Published: September 11, 2024

Cite as: Alfian S, Thurfah J, Griselda M, Puspitasari I. Sleep Disturbances and Depression Levels among General Indonesian Population: A National Survey. Clin Pract Epidemiol Ment Health, 2024; 20: e17450179326359.
http://dx.doi.org/10.2174/0117450179326359240903045716

Send Orders for Reprints to reprints@benthamscience.net

Table S1. Sleeping experience questionnaire.

No.	Question	1. Never	2. Rarely	3. Sometimes	4. Often	5. Always
**1**	I had trouble sleeping	1. Very poor	2. Poor	3. Fair	4. Good	5. Very good
**2**	My quality of sleep was...	1. Not at all	2. A little bit	3. Somewhat	4. Quite a bit	5. Very much
**3**	My sleep was refreshing	1. Not at all	2. A little bit	3. Somewhat	4. Quite a bit	5. Very much
**4**	I was satisfied with my sleep	1. Not at all	2. A little bit	3. Somewhat	4. Quite a bit	5. Very much
**5**	I had difficulty falling asleep	1. Not at all	2. A little bit	3. Somewhat	4. Quite a bit	5. Very much
**6**	I had a hard time concentrating because of poor sleep	1. Not at all	2. A little bit	3. Somewhat	4. Quite a bit	5. Very much
**7**	I had problems during the day because of poor sleep	1. Not at all	2. A little bit	3. Somewhat	4. Quite a bit	5. Very much
**8**	I had a hard time getting things done because I was sleepy	1. Not at all	2. A little bit	3. Somewhat	4. Quite a bit	5. Very much
**9**	I felt tired	1. Not at all	2. A little bit	3. Somewhat	4. Quite a bit	5. Very much
**10**	I felt irritable because of poor sleep	1. Not at all	2. A little bit	3. Somewhat	4. Quite a bit	5. Very much

Table S2. Depression symptoms assessment questionnaire.

No.	Question	How often?			
**1**	I was bothered by things that usually don't bother me	1. Rarely or none (≤1 day)	2. Some days (1-2 days)	3. Occasionally (3-4 days)	4. Most of the time (5-7 days)
**2**	I had trouble concentrating in what I was doing	1. Rarely or none (≤1 day)	2. Some days (1-2 days)	3. Occasionally (3-4 days)	4. Most of the time (5-7 days)
**3**	I felt depressed	1. Rarely or none (≤1 day)	2. Some days (1-2 days)	3. Occasionally (3-4 days)	4. Most of the time (5-7 days)
**4**	I felt everything I did was an effort	1. Rarely or none (≤1 day)	2. Some days (1-2 days)	3. Occasionally (3-4 days)	4. Most of the time (5-7 days)
**5**	I felt hopeful about the future	1. Rarely or none (≤1 day)	2. Some days (1-2 days)	3. Occasionally (3-4 days)	4. Most of the time (5-7 days)

No.	Question	How often?			
**6**	I felt fearful	1. Rarely or none (≤1 day)	2. Some days (1-2 days)	3. Occasionally (3-4 days)	4. Most of the time (5-7 days)
**7**	My sleep was restless	1. Rarely or none (≤1 day)	2. Some days (1-2 days)	3. Occasionally (3-4 days)	4. Most of the time (5-7 days)
**8**	I was happy	1. Rarely or none (≤1 day)	2. Some days (1-2 days)	3. Occasionally (3-4 days)	4. Most of the time (5-7 days)
**9**	I felt lonely	1. Rarely or none (≤1 day)	2. Some days (1-2 days)	3. Occasionally (3-4 days)	4. Most of the time (5-7 days)
**10**	I could not get going	1. Rarely or none (≤1 day)	2. Some days (1-2 days)	3. Occasionally (3-4 days)	4. Most of the time (5-7 days)

Table S3. Bivariate analysis of the correlation between sleep disturbance and sociodemographic factors with depression.

No.	Variable	Depression Incidence				n	(%)	p-value
		Yes	(%)	No	(%)			
1.	**Age (Years)**	-	-	-	-	-	-	-
	15-29	59	0.8	7320	99.2	7379	33.5	0.113[a]
	30-49	82	0.8	9727	99.2	9809	44.5	-
	More than 49	55	1.1	4781	98.9	4836	22.0	-
2.	**Gender**	-	-	-	-	-		-
	Female	125	1.1	10171	86.3	11783	53.5	0.005[a]
	Male	71	0.7	11658	113.8	10242	46.5	-
3.	**Body Mass Index**	-	-	-	-	-	-	-
	Malnutrition	13	1.3	975	98.7	988	4.5	0.390
	Skinny	19	1.0	1792	99.0	1811	8.2	-
	Normal	108	0.9	12285	99.1	12393	56.3	-
	Overweight	26	1.0	2654	99.0	2680	12.2	-
	Obesity	30	0.7	4123	99.3	4153	18.9	-
4.	**Marital Status**	-	-	-	-	-	-	-
	Unmarried	34	0.8	4177	99.2	4211	19.1	0.000[a]
	Married	130	0.8	15931	99.2	16061	72.9	-
	Divorced (Still Alive)	17	2.8	584	97.2	601	2.7	-
	Divorced (by Death)	15	1.3	1130	98.7	1145	5.2	-
5.	**Working Status**	-	-	-	-	-		-
	Unemployed	174	0.9	19892	99.1	20066	91.1	0.305
	Employed	22	1.1	1937	98.9	1959	8.9	-
6.	**Last Education**	-	-	-	-	-	-	-
	Elementary School	96	1.4	6894	98.6	6990	33.4	0.000[a]
	Junior High School	19	0.4	4213	99.6	4232	202.5	-
	Senior High School	47	0.7	6683	99.3	6730	322.0	-
	University	20	0.7	2937	99.3	2957	141.5	-
7.	**Residence Location**	-	-	-	-	-		-
	Rural	99	1.1	12964	144.6	8964	40.7	0.006[a]
	Urban	97	0.7	8865	67.9	13061	59.3	-
8.	**Province**	-	-	-	-	-	-	-
-	Nanggroe Aceh Darussalam	0	0.0	1	100.0	1	0.0	0.003[a]
	North Sumatra	1	4.5	21	95.5	22	0.1	-
	West Sumatra	0	0.0	8	100.0	8	0.0	-
	Riau	0	0.0	7	100.0	7	0.0	-
	Jambi	0	0.0	18	100.0	18	0.1	-
	South Sumatra	0	0.0	33	100.0	33	0.2	-
	Lampung	0	0.0	18	100.0	18	0.1	-
	Kepulauan Bangka Belitung	0	0.0	13	100.0	13	0.1	-
	Kepulauan Riau	1	4.8	20	95.2	21	0.1	-
	DKI Jakarta	6	1.1	552	98.9	558	2.7	-
	West Java	50	1.3	3663	98.7	3713	17.8	-
	Central Java	32	0.9	3724	99.1	3756	18.0	-
	DI Yogyakarta	4	0.3	1567	99.7	1571	7.5	-
-	East Java	13	0.3	3739	99.7	3752	18.0	-

No.	Variable	Depression Incidence				n	(%)	p-value
		Yes	(%)	No	(%)			
-	Banten	8	0.9	853	99.1	861	4.1	-
-	Bali	11	0.8	1407	99.2	1418	6.8	-
-	West Nusa Tenggara	27	1.2	2160	98.8	2187	10.5	-
-	West Kalimantan	0	0.0	4	100.0	4	0.0	-
-	Central Kalimantan	1	3.0	32	97.0	33	0.2	-
-	South Kalimantan	12	0.9	1350	99.1	1362	6.5	-
-	East Kalimantan	0	0.0	85	100.0	85	0.4	-
-	South Sulawesi	16	1.1	1392	98.9	1408	6.7	-
-	West Sulawesi	0	0.0	51	100.0	51	0.2	-
-	West Papua	0	0.0	2	100.0	2	0.0	-
9.	**Sleep Disturbance**	-	-	-	-	-		-
	Severe	44	6.6	627	93.4	671	3.0	0.000 [a]
	Moderate	86	2.4	3557	97.6	3643	16.5	-
	Mild	36	0.7	5105	99.3	5141	23.3	-
	None to slight	30	0.2	12540	99.8	12570	57.1	-
		-	-	-	-	-	-	-

Note: [a] significant factor ($p < 0.05$).
